# A review on computational systems biology of pathogen–host interactions

**DOI:** 10.3389/fmicb.2015.00235

**Published:** 2015-04-09

**Authors:** Saliha Durmuş, Tunahan Çakır, Arzucan Özgür, Reinhard Guthke

**Affiliations:** ^1^Computational Systems Biology Group, Department of Bioengineering, Gebze Technical University, KocaeliTurkey; ^2^Department of Computer Engineering, Boǧaziçi University, IstanbulTurkey; ^3^Leibniz Institute for Natural Product Research and Infection Biology – Hans-Knoell-Institute, JenaGermany

**Keywords:** pathogen–host interaction, computational systems biology, bioinformatics, omics data, protein–protein interaction, metabolic interaction, gene regulatory network, drug target

## Abstract

Pathogens manipulate the cellular mechanisms of host organisms via pathogen–host interactions (PHIs) in order to take advantage of the capabilities of host cells, leading to infections. The crucial role of these interspecies molecular interactions in initiating and sustaining infections necessitates a thorough understanding of the corresponding mechanisms. Unlike the traditional approach of considering the host or pathogen separately, a systems-level approach, considering the PHI system as a whole is indispensable to elucidate the mechanisms of infection. Following the technological advances in the post-genomic era, PHI data have been produced in large-scale within the last decade. Systems biology-based methods for the inference and analysis of PHI regulatory, metabolic, and protein–protein networks to shed light on infection mechanisms are gaining increasing demand thanks to the availability of omics data. The knowledge derived from the PHIs may largely contribute to the identification of new and more efficient therapeutics to prevent or cure infections. There are recent efforts for the detailed documentation of these experimentally verified PHI data through Web-based databases. Despite these advances in data archiving, there are still large amounts of PHI data in the biomedical literature yet to be discovered, and novel text mining methods are in development to unearth such hidden data. Here, we review a collection of recent studies on computational systems biology of PHIs with a special focus on the methods for the inference and analysis of PHI networks, covering also the Web-based databases and text-mining efforts to unravel the data hidden in the literature.

## Introduction

Infectious diseases are one of the preliminary causes of death worldwide each year. Emerging and reemerging diseases and drug resistant pathogens have made the problem more serious for human beings. Therefore, novel therapeutic strategies, called theranostics, are increasingly investigated to fight the biological threats. These strategic solutions require a systems biological approach with a thorough understanding of the underlying mechanisms of infections by focusing on molecular interactions between pathogenic and host organisms (Morens et al., 2004; Murali et al., 2011; Guthke et al., 2012; Durmuş Tekir and Ülgen, 2013). Systems biology is an interdisciplinary research field in life sciences focusing on the study of non-linear interactions among biology entities through the integration and combination of biomolecular and medical sciences with mathematical, computational, and engineering disciplines (Kitano, 2002). By modeling biological phenomena, systems biology uses a more holistic approach based on omics data instead of the traditional reductionism focusing at only a few molecules and interactions. The pathogen–host interactions (PHIs) may be between proteins, nucleotide sequences, metabolites, and small ligands. The protein–protein interactions (PPIs) have been identified as the most important type in the functioning of PHI systems and therefore are the most studied type (Stebbins, 2005; Korkin et al., 2011; Zoraghi and Reiner, 2013). However, non-coding RNAs (ncRNAs) and metabolites have also been reported to have critical functional roles in virus–host and bacteria–host interactions, respectively (Gottwein and Cullen, 2008; Skalsky and Cullen, 2010; Eisenreich et al., 2013; Saayman et al., 2014).

Different levels of omics data collected from pathogens and/or infected cells are crucial components that drive bioinformatic analyses facilitating the construction and analysis of infection-specific gene-regulatory, metabolic, and protein–protein networks (Westermann et al., 2012; Schulze et al., 2015). Such network-based computational systems biology analyses of PHI-based omics data enable the elucidation of infection mechanisms and their dynamics, the identification of potential drug targets for the next-generation antimicrobial therapeutics, and the development of novel and personalized strategies for the prevention and treatment of infections. With an increasing amount of experimental PHI data, Web-based databases were developed to derive and provide pathogen–host interactome data, usually focusing on specific pathogens or hosts (Wattam et al., 2014; Ako-Adjei et al., 2015; Calderone et al., 2015; Guirimand et al., 2015). Although the available databases are promising in data archiving, a huge amount of PHI data is not stored in any of these databases, since these data are buried in the literature. Therefore, there is an urgent need for novel text mining methods specific for PHI data retrieval. In this paper, the efforts on the collection of PHI-based omics data are reviewed first. Next, a review of the computational systems biology analyses of three major types of PHI networks is provided. Then, the available PHI databases and the current snapshot of the literature on text mining for PHI data are presented.

## Omics Data Reflecting PHI Networks

The systems biology approaches with genome-wide molecular profiling using high-throughput techniques to generate omics data are changing the face of infection biology together with the computational methods for heterogeneous data management and integrative analysis via mathematical modeling (Guthke et al., 2012; Law et al., 2013). New insights in the microbial and viral pathogenesis, in particular in the host’s immune response to contact with pathogens, offer opportunities for better diagnostics, therapeutics, and vaccines. Thus, systems biology of infection allows to yield novel therapeutic targets (Sarker et al., 2013) and to establish individualized or personalized medicine. The integrative personal omics profile (iPOP) combines genomics, transcriptomics, proteomics, metabolomics, and autoantibody profiles from a single individual over a 14-month period (Chen et al., 2012; Li-Pook-Than and Snyder, 2013).

There are various platforms for handling of measured data from samples, data storage and exchange, data pre-processing and data analysis. Powerful platforms for data management in systems biology have recently become available and are standardized step by step by the Functional Genomics Data Society^1^ (FGED, founded in 1999 as MGED; Brazma et al., 2006). Several systems biology projects in Europe including the ones dedicated to PHI research use the SysMO-DB/SEEK system for sharing data, knowledge (including Standard Operating Procedures – SOPs) and mathematical models^2^ (Wolstencroft et al., 2011). For the management of genomics, transcriptomics, and (2D-gel) proteomics data in infection research, the data warehouse ‘OmniFung’ was established to support research on fungi–host interactions^3^ (Albrecht et al., 2011, 2007).

The free, open source and open development software project Bioconductor, which is primarily based on the statistical R programming language, provides 934 software packages, 894 annotation and 224 experimental data sets for the bioinformatic analysis and comprehension of high-throughput genomic data^4^ (Version 3.0). These packages as well as other R packages not included in the Bioconductor project are useful for the advanced, in particular integrative, analysis of omics data and modeling of PHIs. To identify genes, proteins or metabolites of interest for biomarker discovery or drug target prediction by supervised machine learning methods, there are many data mining tools available. For instance, WEKA^5^ or RapidMiner^6^ is used to characterize the response of the host immune system by decision tree analysis of flow cytometric data (Simon et al., 2012). In addition, there are platforms and software tools for the integrative and explorative analysis and visualization of data from the different omics levels of PHIs (Horn et al., 2014).

### PHI-Based Genome and Transcriptome Data

The genomic information from the host and the pathogen represents the basis for all further molecular analyses and bioinformatic investigations of PHI systems. Thus, genome sequencing is fundamental. It helps to improve diagnosis, typing of pathogen, virulence and antibiotic resistance detection, and development of new vaccines and culture media. Single nucleotide polymorphism (SNP) typing is important for both identification and characterization of variants of pathogens (strains, clinical isolates) as well as to study the susceptibility of humans for certain infections. In the last decade, there was, and in the future there will be, an explosion of genome sequence data. The new sequencing technologies enable small research units to create huge genome datasets at low cost in short time. As a result, handling, comparing, and extracting useful information from millions of sequences becomes more and more challenging, i.e., increased efforts in computational biology are urgently needed. In particular, sequencing is used for genomic and transcriptomic characterization of new emerging pathogens. Whole-genome sequencing based phylogenetic studies have implications for understanding the evolution of the PHIs as well as tracking and possibly preventing infection diseases as performed for the Enterotoxigenic *Escherichia coli* (ETEC), a major cause of infectious diarrhea (von Mentzer et al., 2014). Metagenomic and metatranscriptomic studies of pathogens revealed how pathogenic microorganisms adapt to hosts, e.g., plants (Guttman et al., 2014).

The first step of genome sequence analysis, the assembling of genome sequence data into a single genomic contig, may be difficult, in particular due to assembling repeated sequences if reference genomes are not available. Then, additional information may be required to resolve the remaining DNA regions. The next step, the functional annotation of virulence-relevant pathogens and focusing on host-interaction genes, is often difficult as the genes of interest for PHIs are frequently species-specific and, thus, studies of gene homologies may not be helpful. The situation would be improved by the databases of protein families involved in host interactions, which incorporate the currently used gene names, sequence motifs, gene functions, and experimental results (see section “Web-Based Databases for PHI Systems”). On the other hand, comparative genomics can provide insights into molecular pathogenesis, host specificity, and evolution of pathogens. Next generation sequencing (NGS) has revolutionized the molecular investigation of the diversity of pathogens on the genomic and transcriptomic level. It enables an efficient analysis of complex human micro-floras, both commensal and pathological, through metagenomic methods. Genomic sequences and their annotations are provided through several portals, such as the Genomes Online Database^7^.

In contrast to the static information from the genome, the transcriptome reflects the dynamics of PHI systems that results in temporal profiles of gene expression with changes in the scale of minutes and hours. More and more, beside the protein-coding mRNAs, also various non-conding small RNAs are investigated. For instance, in *Staphylococcus aureus*, a leading pathogen for animals and humans, about 250 regulatory RNAs were found (Guillet et al., 2013). Repositories for transcriptome data, such as Gene Expression Omnibus^8^ (GEO) and ArrayExpress^9^ freely distribute microarray and NGS (RNA-Seq) data as well as other forms of high-throughput functional genomics data. In GEO, data from more than 1600 organisms, both pathogens and hosts, are accessible. For instance, for the pathogens *Mycobacterium tuberculosis*, *S. aureus*, *Candida albicans*, and *Helicobacter pylori* transcriptome data from 1,855, 1,777, 1,627, and 1,284 samples are available, respectively. Other data sets monitor the transcriptome of the host’s response, e.g., *Homo sapiens* and *Mus musculus* (GSE56091, GSE56093). Some monitor data from host and pathogen simultaneously, e.g., *S. aureus* and the zebrafish *Danio rerio* (GSE32119). NGS has opened the door for simultaneous transcriptome analysis by the so-called dual RNA-Seq (Tierney et al., 2012a,b; Westermann et al., 2012; Camilios-Neto et al., 2014; Longo et al., 2014; Pittman et al., 2014; Xu et al., 2014; Schulze et al., 2015).

### PHI-Based Proteome and Metabolome Data

Proteins are key players in PHIs, in particular in pathogen recognition as well as innate and adaptive immune responses. Pathogen-associated molecular patterns (PAMPs) are molecules or small molecular motifs within a group of pathogens (e.g., the protein flagellin, lipopeptides, lipopolysaccharide – LPS) that are recognized by proteins, the so-called pattern recognition receptors (PRRs), such as Toll-like receptors (TLRs; Qian and Cao, 2013). For instance, TLR4 recognizes bacterial LPS, and TLR5 recognizes bacterial flagellin. The PRRs stimulate signal transduction via pathways, e.g., the tumor necrosis factor alpha (TNFα) signaling or the interferon-gamma (IFNγ)-receptor pathway including the JAK-STAT-pathway. IFNγ is a cytokine that is a key player in innate and adaptive immunity against viral, as well as some microbial and protozoan infections. The nuclear factor NF-κB is a protein, a transcription factor, that is activated by various intra- and extra-cellular stimuli such as bacterial or viral products, for instance via the TLRs signaling and induces the expression of pro-inflammatory cytokines (interleukines, TNFα, Type I interferones). Thus, the application of proteomics is crucial in the investigation of PHI systems and for the above mentioned iPOP, e.g., the immune profiling of patients (Chen et al., 2012).

By dedicated bioinformatic pipelines, a description of pathogen proteomes and their interactions within the context of human host has a strong impact in both diagnostic and clinical treatment of the patient. In the last few years, several advanced proteomic techniques have been established providing individual proteome charts of both pathogens and hosts, including antimicrobial or antimycotic resistance profiling and immune profiling of the patient. Proteome analysis is hampered by the extremely divergent biochemical properties of the individual proteins, making an entire view of the proteome almost impossible (Otto et al., 2014). The coupling of multidimensional separations with mass spectrometry (MS) for protein and peptide analyses via, for instance, the matrix-assisted laser desorption ionization (MALDI) and electrospray ionization (ESI) techniques resulted in powerful MS instrumentations. Many of these MS-based techniques, e.g., MALDI-TOF, have been used in clinical microbiology and research (Del Chierico et al., 2014; Otto et al., 2014). For PHI analyses, the cell wall proteins and the secretomes are of special interest to study the PAMPs and PRRs as well as their interplay (Schmidt and Völker, 2011; Zheng et al., 2011; Heilmann et al., 2012; Di Carli et al., 2012). PHI analysis studies that focus on the host side studying the immune response (Hartlova et al., 2011; Heyl et al., 2014) or on the pathogen side (Bröker and van Belkum, 2011; Cash, 2011; Ahmad et al., 2012) have also been conducted. The integrative analysis of proteome data with other omics data for both pathogens and hosts is a very challenging task in bioinformatics (Albrecht et al., 2010, 2011).

Stanberry et al. (2013) demonstrated on the host side a strong association between the metabolome profiles, i.e., the metabolite expression levels of differentially expressed pathways, and their temporal patterns at each time point with the disease status of viral infection with a human rhinovirus and a respiratory syncytial virus. For metabolic studies on the pathogen side, there are *in silico* strategies to identify effective targets for anti-infective drugs based on constraint-based modeling of genome-scale metabolic networks (Chavali et al., 2012; see section “PHI Metabolic Network Models”). A prominent type of PHIs is the production of toxins by the pathogens that attack the host. For instance, gliotoxin produced by the human-pathogen fungus *Aspergillus fumigatus* modulates the immune response and induces apoptosis in the host (Gardiner and Howlett, 2005; Scharf et al., 2012). Another type of PHI is due to the pathogens that frequently utilize substrates from the host (Rohmer et al., 2011). The gene regulatory network (GRN) model-assisted studies of the uptake of essential substrates such as iron (Linde et al., 2010, 2012) or nitrogen sources (Ramachandra et al., 2014) by such pathogens address specific but important aspects of PHIs.

## Computational Systems Biology of PHI Networks

A systems biology approach is crucial to model and understand PHIs, in particular interactions between the immune system of humans or animals, and the pathogens (Berglund et al., 2009; Guthke et al., 2012; Horn et al., 2012; Zhou et al., 2013). Systems biology of PHIs aims at describing and analyzing the confrontation of the host with viral, bacterial, and fungal pathogens and parasites by the development of testable computational models of PHIs. The predictive power of such models enables diagnosis and therapy by the prediction of biomarkers and drug targets. Systems biology of PHIs includes an integrative analysis and modeling of genome-wide and/or spatio-temporal data from both the host and the pathogen, or the response of the host or pathogenic cells to defined perturbations that simulate conditions during infection.

At the computational side, systems biology of PHIs comprises:

– Modeling of molecular mechanisms of infections,
– Modeling of non-protective and protective immune defenses against pathogens to generate information for possible immune therapy approaches,
– Modeling of PHI dynamics and identification of biomarkers for diagnosis and for individualized therapy of infections,
– Identifying essential virulence determinants and host factors, and thereby predicting potential drug targets
– Understanding of PHIs, in particular the immune system and the immune evasion of the pathogens, as the result of evolutionary long-term adaptation and selection.

Both the innate and the adaptive immune system comprise cell-mediated and humoral components. Thus, systems biology of immune defense has to handle multi-scale modeling from molecular to systemic/organ level. The same is required for the pathogen side. The interaction of cellular components is preferentially the area of the agent-based modeling, whereas the humoral immunity can be modeled by ordinary differential equations (ODEs). While the innate immune response is non-specific and acts immediately, the adaptive immune response is pathogen and antigen specific with time lag and immunological memory. Thus, the temporal organization and population dynamics have to be modeled in a different manner for the innate and adaptive immune system in interaction with the pathogen (Perelson, 2002; Gottschalk et al., 2013; Six et al., 2013; Panayidou et al., 2014).

The study of the interplay between pathogens and immune cells remains a challenging task due to its complexity. While the emerging image-systems biology of cellular interaction (Mech et al., 2011; Hünniger et al., 2014; Kraibooj et al., 2014; Pollmächer and Figge, 2014) is here out of the scope, the present review focuses on the molecular, mainly omics data-based level. Here, a difficulty arises to separate host’s transcripts, proteins, and metabolites from that in the pathogen and to extract them in a balanced amount for a simultaneous monitoring of these molecules so that the network models of PHIs are inferred. Therefore, most studies focus either on the pathogen or the host side with a defined and controlled change of the respective other side as an external perturbation, i.e., considering an input from the outside of the investigated system. Thus, to simplify the study, the PHIs have been studied mainly in one direction either from pathogen to host or from host to pathogen. Only very recently, the bi-directional interaction of pathogen and host became observable simultaneously using the so-called dual RNA-Seq data generated by NGS of the transcriptome of pathogen and host (see section “PHI-Based Genome and Transcriptome Data”).

Understanding the evolutionary dynamics of PHIs by mathematical modeling in terms of both molecular mechanisms and selective forces is important in order to design drugs that will be effective in the long term, i.e., to avoid or to overcome resistance to antibiotics (Guo et al., 2011; Lima et al., 2013; Palmer and Kishony, 2013). Finally, computational systems biology approaches are and will be used to select pathogen-host drug targets and to develop novel anti-infectives and vaccines (Brown et al., 2011; Mooney et al., 2013; Sarker et al., 2013; Rienksma et al., 2014).

### PHI Regulatory Network Models

Biological network models are widely used to improve our understanding of infectious diseases (Mulder et al., 2014). There are many small-scale models (mainly ODE-based), which describe PHIs phenomenologically (Baccam et al., 2009; Saenz et al., 2010; Manchanda et al., 2014). These models without molecular specification are out of the scope of this review, as they usually do not predict PHIs on the molecular level. Here, omics data based PHI models will be reviewed.

**FIGURE 1:**
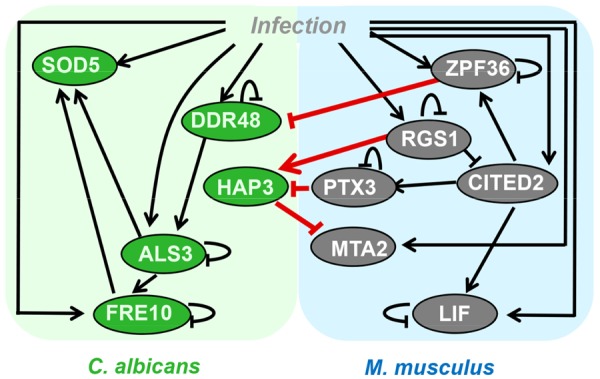
**Network model describing pathogen-host interactions between *C. albicans* and murine dendritic cells based on dual RNA-Seq data (modified from Tierney et al., 2012b)**.

Computational modeling of GRNs reveals the molecular logic of adaptation of pathogens to their hosts, the immune evasion of the pathogen as well as the immune response of the host to infection with pathogens. GRNs provide causal explanations for the differentiation, the developmental and effector states, as well as the fate dynamics of immune cells (Singh et al., 2014). Finally, GRNs may also describe the interaction of the two networks, one of the pathogen and the other of the host (see **Figure 1** for example). The inference of GRN models from gene expression data is a problem of great importance for PHI studies. Various reverse engineering methods have been proposed, which include methods based on Boolean networks, Bayesian networks, differential or difference equations, and graphical Gaussian models. In general, due to the high dimensionality (thousands of genes and proteins in both host and pathogen organisms) versus the limited number of samples (not more than hundreds in the case of steady state data from knock-out (KO) mutants; only a few samples in *in vivo* studies of PHI monitored at, e.g., 5–10 time points), the GRN inference is underdetermined implying that there could be many equivalent (indistinguishable) solutions. Motivated by this fundamental limitation, there are various approaches for GRN inference. Again, there are outstanding review articles covering the long-standing problem of gene expression data-driven GRN inference (De Jong, 2002; van Someren et al., 2002; Gardner and Faith, 2005; Bansal et al., 2007; Emmert-Streib et al., 2014; Linde et al., 2015). One of the conclusions from the DREAM initiative^10^ (Dialog for Reverse Engineering Assessment of Methods; Prill et al., 2010) that performed a comprehensive blind assessment of over 30 network inference methods was that no single inference method performs optimally across all datasets. Integration of predictions from multiple inference methods shows more robustness and higher performance across diverse datasets (Marbach et al., 2012). For instance, the algorithm TRaCE performs an ensemble inference of GRNs, which takes into account the inherent uncertainty associated with discriminating direct and indirect gene regulations from steady-state data of KO experiments (Ud-Dean and Gunawan, 2014). Another group of GRN inference approaches includes prior knowledge as reviewed by (Hecker et al., 2009; Isci et al., 2014) or further experimental data (Greenfield et al., 2010). A third group of GRN algorithms restricts the GRN to static networks inferred from steady state data (e.g., from KO mutants of the pathogen) or to small-scale networks with a few nodes (genes, proteins), where the pre-selection of them is the critical point (Nakajima and Akutsu, 2014).

The genome-wide GRN model inference, when restricted to the static network models of thousands of genes, requires large gene expression data sets and prior-knowledge in high quality and quantity, which is not the case for most of the pathogens of interest as demonstrated for the human-pathogen *C. albicans* (Altwasser et al., 2012). In contrast to the genome-wide GRN models, the small-scale network models that take into account 5–50 genes or proteins are often used for PHI studies. These models do not represent the holistic view as it is claimed in systems biology, but they generate hypotheses of PHIs that drive further experimental work in infection biology. Afterward, the GRN-based *in silico* predictions have to be validated experimentally. This approach of focused small-scale GRN inference was reported particularly for human–pathogen fungal infection (Linde et al., 2010, 2012; Ramachandra et al., 2014) by using the ODE-based NetGenerator algorithm. The algorithm was primarily introduced to model the immune response to bacterial infection (Guthke et al., 2005; Weber et al., 2013). This algorithm was also applied for the inference of the PHIs of the human–pathogen fungus *C. albicans* with murine dendritic cells based on dual RNA-Seq data (Tierney et al., 2012b). Here for instance, based on the inferred GRN model shown in **Figure 1**, an inhibition of the expression of the protein HAP3 in the fungus by the murine pentraxin (PTX3) was computationally predicted and, afterward, experimentally validated.

### PHI Metabolic Network Models

Pathogens are dependent on the host environment for the substrates required to maintain a metabolically active state (Chavali et al., 2012; Eisenreich et al., 2013). Therefore, the exchange of several metabolites takes place between pathogens and their host. Besides, the production of virulence factors by the pathogen requires energy, and, hence, an active metabolism, making the nutrients in the host environment crucial for the infection to occur (Milenbachs et al., 1997). The direct functional link between metabolism and virulence is also supported by the finding that metabolic and virulence genes are located on the same pathogenicity island for some pathogens (Rohmer et al., 2011; Heroven and Dersch, 2014). In a different approach, the authors used a network-based computational analysis to elucidate common targeting strategies of bacteria and viruses on human (Durmuş Tekir et al., 2012), based on pathogen–host PPIs stored in the PHISTO database (Durmuş Tekir et al., 2013). Their results revealed metabolism as a common strategy of both pathogen types to target human cells. The role of metabolism in the pathogenesis was also emphasized by others (Kafsack and Llinás, 2010). Therefore, metabolism is a candidate target for anti-microbial therapies.

There are well-established bioinformatic methods for metabolic network reconstruction, based on DNA genome sequences and constraint based modeling covered by outstanding review articles (Feist et al., 2008; Oberhardt et al., 2009; Ruppin et al., 2010; Bordbar and Palsson, 2012). The *in silico* methods for metabolic network reconstruction are highly valuable for understanding the physiology of the pathogen, e.g., the biosynthesis of toxins that attack the host or the substrate requirement that shows the dependency of the pathogen on the environment within the host. At the host side, the human metabolic network reconstruction may also have an impact for drug discovery and development (Ma and Goryanin, 2008). A systematic modeling of the metabolic trafficking between pathogens and its hosts first started with the constraint-based modeling of the Gram-negative bacterial pathogen, *Salmonella typhimurium* (Raghunathan et al., 2009). The authors reconstructed a genome-scale metabolic model for the pathogen in question, and then simulated its survival capabilities with the flux-balance approach (Kauffman et al., 2003; Orth et al., 2010). When they used a media mimicking host-cell nutrient environment (e.g., macrophage) rather than laboratory media, their correct predictions considerably increased. They also showed that the use of gene expression data can lead to a better inference of active transport mechanisms, and hence the host cell environment. In another study, the reconstructed metabolic network of the malaria-causing protozoan parasite, *Plasmodium falciparum*, was embedded into its host, erythrocyte, and the combined pathogen-host network was simulated via flux-balance analysis (FBA; Huthmacher et al., 2010). The novelty here was to take also the host network into account to predict metabolite exchanges between the parasite and the host, rather than only considering the host environment to account for pathogen–host metabolic interactions. Such a consideration is important since a pathogen infection causes pathogen-specific or common responses in the host metabolic pathways from central carbon metabolism to fatty acid and amino acid metabolisms (Eisenreich et al., 2013). Their analysis resulted in the prediction of antimalarial drug targets (Huthmacher et al., 2010).

In a more systematic study, genome scale metabolic networks of *Mycobacterium tuberculosis* and its host, alveolar macrophage, were reconstructed in an integrated fashion and the integrated pathogen-host metabolic model was used to analyze infection mechanisms and related different pathological states (Bordbar et al., 2010). The reconstructed joint metabolic network covered 2071 genes (661 for the pathogen, 1410 for the macrophage), controlling a total of 4489 reactions. Integrative analysis of the network with the transcriptome data from the infected macrophage cells enabled the inference of the induced changes in the pathogen. One important issue in the network based drug-target identification is the selectivity of the identified targets. The candidate target must make no harm to the host. This was taken into consideration by (Bazzani et al., 2012), where they used the integrated pathogen-host metabolic model of *Plasmodium falciparum* and hepatocyte, the first human infection site for malaria parasites. The flux balance approach was combined with 48 experimental antimalarial drug targets to identify the targets which are essential for the parasite but not essential for hepatocyte metabolism. The *in silico* analysis led to the ranking of the identified targets with respect to their reducing effect on the cellular fitness.

One key point in the elucidation of metabolic mechanisms both in the host and in the pathogen is to correctly characterize the nutrient availability for the pathogen in the host environment. This characterization is also important for successful modeling attempts. The available nutrients shape the active parts of the pathogen metabolism, and also the depletion of different metabolites may trigger different responses in the host (Bumann, 2009; Rohmer et al., 2011; Eisenreich et al., 2013; Sasikaran et al., 2014). Therefore, nutritional environment has a crucial role to understand the basis of infection mechanisms (Brown et al., 2008; Gouzy et al., 2014). Systems-level experimental approaches such as lipidomics and metabolomics are getting popular to decipher the pathogen–host nutritional interactions (Wenk, 2006; Olszewski et al., 2009; Antunes et al., 2011). A recent attempt to identify active metabolic routes from the host environment to pathogen inside by using 13C flux spectral analysis (Beste et al., 2013) provided a quantitative measure of interactions between *Mycobacterium tuberculosis* and its host macrophage. The experimental labeling data enabled the identification of substrates used by the pathogen. Another elegant study used 13C-labeling based fluxomics as well as metabolomics and proteomics to shed light on the metabolic interplay between *Shigella flexneri* and HeLa epithelial cells (Kentner et al., 2014). They were able to identify host metabolites that contribute to the growth of Shigella as substrates.

Similar to the use of gene expression data to infer GRNs as discussed in the previous section, metabolome data obtained from the infected cells or PHI systems can be used to infer infection-specific metabolic networks by using reverse engineering approaches. Taking into account several bioinformatics methods proposed for this type of inference as reviewed recently (Cakir and Khatibipour, 2014), we believe the field of infection will witness promising applications in the coming years.

### PHI Protein–Protein Network Models

In the post-genomic era, genes and the corresponding proteins are studied thoroughly, allowing the identification of intra- and interspecies protein interaction networks. Following the development of experimental techniques to produce large-scale molecular interaction data (Fields and Song, 1989; Fisher et al., 2002; Gavin et al., 2002; Ho et al., 2002), the first large-scale intraspecies PPIs were produced experimentally (Finley and Brent, 1994; Bartel et al., 1996; Fromont-Racine et al., 1997; Flajolet et al., 2000; Ito et al., 2000; McCraith et al., 2000; Walhout et al., 2000; Rain et al., 2001). On the other hand, the initial efforts to identify large scale interspecies protein interaction data for PHI systems have been performed since 2007 (**Table 1**). The first large scale PHI examples were for commonly observed and human-threatening viruses and bacteria. These were firstly for viral pathogens; Epstein-Barr virus (EBV; Calderwood et al., 2007; Forsman et al., 2008), Hepatitis C virus (HCV; De Chassey et al., 2008; Tripathi et al., 2010; Dolan et al., 2013; Ngo et al., 2013), Human Immunodeficiency Virus (HIV; Gautier et al., 2009; Jäger et al., 2012), Influenza A virus (Shapira et al., 2009), Dengue virus (DENV; Khadka et al., 2011), Measles virus (MV; Komarova et al., 2011), and Human Respiratory Syncytial Virus (HRSV; Wu et al., 2012). On the other hand, the large scale experimental detection of bacteria-human protein interaction networks was performed for *Bacillus anthracis*, *Francisella tularensis*, and *Yersinia pestis* (Dyer et al., 2010; Yang et al., 2011).

**Table 1:** The large-scale pathogen–human PPI networks in chronological order.

Pathogen name	Pathogen type	Number of PHIs	Number of interacting pathogen proteins	Number of interacting human proteins	Reference
EBV	DNA virus	173	40	112	Calderwood et al. (2007)
HCV	RNA virus	481	11	421	De Chassey et al. (2008)
EBV	DNA virus	147	1	147	Forsman et al. (2008)
HIV-1	Retrovirus	183	1	183	Gautier et al. (2009)
Influenza A virus (H1N1 A/PR/8/34)	RNA virus	135	10	87	Shapira et al. (2009)
Influenza A virus (H3N2 A/Udorn/72)	RNA virus	81	10	66	Shapira et al. (2009)
*Bacillus anthracis*	Gram-positive bacteria	3,073	943	1,748	Dyer et al. (2010)
*Yersinia pestis*	Gram-positive bacteria	4,059	1,218	2,108	Dyer et al. (2010)
*Francisella Tularensis*	Gram-negative bacteria	1,383	349	999	Dyer et al. (2010)
HCV	RNA virus	56	2	56	Tripathi et al. (2010)
DENV	RNA virus	139	10	105	Khadka et al. (2011)
MV	RNA virus	245	1	245	Komarova et al. (2011)
*Y. pestis*	Gram-positive bacteria	204	66	109	Yang et al. (2011)
HIV-1	Retrovirus	497	16	435	Jäger et al. (2012)
30 viral species	DNA and RNA viruses	1681	70	579	Pichlmair et al. (2012)
HRSV	RNA virus	221	1	221	Wu et al. (2012)
HCV	RNA virus	112	7	94	Dolan et al. (2013)
HCV	RNA virus	103	1	103	Ngo et al. (2013)

As an initial large scale virus–human PHI network example, protein interactions between the herpesvirus EBV and human were mapped by the yeast two hybrid (Y2H) method, providing 173 PHIs between 40 EBV proteins and 112 human proteins (Calderwood et al., 2007). EBV is the infectious cause of several human diseases such as Burkitt’s lymphoma, Hodgkin’s disease, and nasopharyngeal carcinoma. This EBV–human protein interaction network enabled the initial observations about EBV strategies (i.e., targeting hub and bottleneck human proteins) for replication and persistence within the host. For the same viral system, 147 human protein interactors for EBV nuclear antigen 5 (EBNA5) were identified with LC-MS/MS in a following study (Forsman et al., 2008). Multifunctional viral protein EBNA5 is already known to be critical in EBV pathogenesis, and these PHI data provided further insights on its molecular mechanisms during infection. The identified interactions between EBNA5 and the human proteins functioning in protein control systems that recognize proteins with abnormal structures may indicate the roles of the viral protein in this system.

The first proteome-wide PHI map for the flavivirus HCV, a major cause of chronic liver diseases, was deduced by Y2H and then by literature mining of previously found interactions between HCV and human, providing a large network for such a small-genome organism. The resulting network consists of 481 interactions between 11 HCV proteins and 421 human proteins. Pathway enrichment analysis of the targeted cellular proteins indicated focal adhesion as a new function subverted by HCV (De Chassey et al., 2008). Using the same experimental approach, 11 human proteins interacting with HCV Core protein and 45 interacting with NS4B (one of the six HCV non-structural proteins) were found (Tripathi et al., 2010). To further understand the mechanisms of the interactions between HCV and human proteins, two extended PPI networks were constructed. These networks are composed of the Y2H-derived interactions and the secondary interactors of the human proteins that interact with the Core and NS4B proteins. Functional analysis of these networks pointed to the human proteins ENO1, SLC25A5, and PXN as potential antiviral targets. ENO1 and SLC25A5 are interaction partners of HCV Core protein. PXN is the first neighbor of both ENO1 and SLC25A5 within the human PPI network. Observing the effects of small interfering RNA (siRNA) knockdown of these host proteins on HCV propagation and replication validated the computational network analysis results (Tripathi et al., 2010). Another Y2H screen resulted in 112 unique interactions between 7 HCV and 94 human proteins (Dolan et al., 2013). DENV is another member of the flaviviruses family, causing the severe human disease dengue hemorrhagic fever. Using the Y2H method, 139 PHIs were detected between 10 DENV proteins and 105 human proteins (Khadka et al., 2011). These two PHI networks of HCV–human by Dolan et al. (2013) and DENV–human by Khadka et al. (2011) were analyzed comparatively and a large overlap was observed between HCV and DENV targets. To determine if the common cellular targets play crucial roles in infections, siRNA experiments were performed and the results revealed the required cellular proteins (CUL7, PCM1, RILPL2, RNASET2, and TCF7L2) for HCV replication (Dolan et al., 2013). Finally, using protein microarray assays, 103 human proteins were identified as HCV Core-interacting partners. Through these PHI data, the viral modulation of some cellular mechanisms was studied in detail and the cellular MAPKAPK3 was proposed as a potential therapeutic target for HCV infections (Ngo et al., 2013). Prior to these studies, a number of small scale PHI data were produced for the HCV–human interaction system (Matsumoto et al., 1997; Hsieh et al., 1998; Lu et al., 1999; Owsianka and Patel, 1999).

Orthomyxovirus Influenza A virus is the source of all flu pandemics infecting multiple species. For H1N1 A/PR/8/34 strain of influenza virus, 135 PHIs were identified between 10 viral and 87 human proteins, most of which are expressed in primary human bronchial cells. For another strain of influenza A virus, H3N2 A/Udorn/72, a PHI network with 81 interactions between 10 viral and 66 human proteins was constructed. Both of the PHI networks were detected by the Y2H method. Similarities of these two PHI networks highlighted the conserved functions of influenza virus proteins through strains. Observing the topological network properties of these Influenza A virus–human PPI networks allowed to draw crucial conclusions on the multi-functionality of the small number of proteins encoded by RNA viruses, revealing that viral proteins can interact with a significant number of human proteins (Shapira et al., 2009).

AIDS-causing retrovirus HIV, probably the most studied human pathogen, depends largely on human cellular machinery to be replicated, like other RNA-carrying viruses. One large-scale PHI dataset for HIV-1 was produced using affinity chromatography coupled with MS, resulting in 183 human nuclear proteins as interacting partners of HIV-1 Tat (nuclear regulatory protein) which is essential for viral replication within the host nucleus. The following *in silico* analysis of the experimentally verified PHI data provided further insights on the mechanisms of Tat during HIV-1 infection. Firstly, motif composition analysis highlighted that Tat-targeted cellular proteins are enriched for domains mediating protein, RNA and DNA interactions, and helicase and ATPase activities. Secondly, functional analysis of Tat-targeted human proteins showed that they are enriched for a wide range of biological processes such as gene expression regulation, RNA biogenesis, chromatin structure, chromosome organization, DNA replication, and nuclear architecture (Gautier et al., 2009). Another large PHI network was constructed for HIV–human protein complexes by affinity tagging and purification MS, resulting in 497 PHIs between 16 HIV-1 proteins and 435 human proteins. In that study, the functional categories of HIV-targeted human proteins were analyzed indicating that the host factors in the found PHI network are enriched for the transcription and the regulation of ubiquitination. Additionally, the domains of the interacting proteins were also investigated, and the enriched domain types (14-3-3 domains and β-propellers) in targeted human proteins were identified to facilitate future structural modeling studies (Jäger et al., 2012). For HIV-1, several small scale experiments were also carried out to find protein PHI data (Cujec et al., 1997; Le Rouzic et al., 2002; BonHomme et al., 2003; Lusic et al., 2003; Naji et al., 2012) establishing HIV-1 as the pathogenic species having the largest experimentally verified PHI data.

Using the approach of combining modified tandem affinity chromatography and MS analysis, 245 cellular interacting proteins were identified for the viral protein MV-V (one of the virulence factors of paramyxovirus MV). MV-V was found to target known key components of the host antiviral response including STAT1, STAT2, IFIH1, and p53, and also essential components of ribosome, reticulum, and mitochondria. The topological and functional analysis of human proteins targeted by MV-V shows that they have properties within the human interactome similar to the well-known targets of other viruses (Komarova et al., 2011).

As an example for another multi-functional viral protein, HRSV (another member of paramyxoviruses) NS1 can act as an antagonist of host type I and III interferon production and signaling, inhibit apoptosis, suppress dendritic cell maturation, control protein stability, and regulate transcription of host cell mRNAs, among its other functions. A total of 221 PHIs were determined between only one viral protein NS1 and human proteins, reflecting its multifunctional nature. This virus-human PHI network was produced by quantitative proteomics in combination with green fluorescent protein (GFP)-trap immunoprecipitation. It was observed that many of the HRSV-targeted human proteins have roles in transcriptional regulation and cell cycle regulation (Wu et al., 2012).

A study covering several DNA and RNA viruses (Pichlmair et al., 2012) found 1681 PHIs between 70 viral ORFs from 30 species and 579 human proteins. The interacting cellular proteins were isolated by tandem affinity purification (TAP), and the purified proteins were analyzed by one-dimensional gel-free liquid chromatography tandem MS (LC–MS/MS). A comparative interactomics analysis of the produced viral PHI networks (DNA viruses versus RNA viruses) provided crucial insights on the infection strategies of DNA and RNA viruses. It was concluded that RNA viruses target the JAK–STAT and chemokine signaling pathways, as well as pathways associated with intracellular parasitism, whereas DNA viruses target cancer pathways (Pichlmair et al., 2012).

The first extensive bacterial PHI networks were identified for important human pathogens, *B. anthracis*, *F. tularensis*, and *Y. pestis* (Dyer et al., 2010; Yang et al., 2011). Gram-positive bacteria *B. anthracis* and *Y. pestis* and Gram-negative bacterium *F. tularensis* are respiratory pathogens causing anthrax, bubonic plague, and acute pneumonic disease, respectively. Using the Y2H method, large-scale interaction data were generated between these bacteria and human, leading to 3073 PHIs between 943 *B. anthracis* proteins and 1748 human proteins, 4059 PHIs between 1218 *Y. pestis* proteins and 2108 human proteins, and 1383 PHIs between 349 *F. tularensis* proteins and 999 human proteins. Bioinformatic analysis of these experimentally found bacteria–human interaction data revealed that bacterial proteins preferentially interact with human proteins that are hubs and bottlenecks in the human PPI network, as previously observed for viral PHIs. The modules of bacterial PHIs that are conserved amongst the three networks were computed. The found conserved modules may reveal commonalities among how different bacterial pathogens interact with crucial host pathways involved in inflammation and immunity (Dyer et al., 2010). A different Y2H strategy was used for *Y. pestis* by choosing only potential virulence factors as bait proteins. 204 PHIs were identified between 66 *Y. pestis* proteins and 109 human proteins, and then 23 previously reported PHIs were integrated to construct a comprehensive network between *Y. pestis* and human (Yang et al., 2011).

The increase in the amount of experimentally verified pathogen–human PPI data allowed a number of bioinformatic studies to investigate infection mechanisms at the level of PHIs for different pathogen types (Dyer et al., 2008; Singh et al., 2010; Durmuş Tekir et al., 2012). The first global analysis of more than 10,000 PHI data revealed important observations (Dyer et al., 2008). Firstly, targeting hub and bottleneck proteins were concluded as a common behavior for all pathogens. Targeting human transcription factors and key proteins that control the cell cycle and regulate apoptosis and transport of genetic material across the nuclear membrane were found to be common infection strategies of viruses. On the other hand, targeting human proteins that function in the immune response was observed as a common bacterial infection strategy (Dyer et al., 2008). In a following study, investigation of more than 20,000 experimental PHI data revealed that the preference of interacting with hub and bottleneck proteins is more pronounced in viruses than bacteria. The analysis of the human proteins targeted by both bacteria and viruses indicated that attacking human metabolic processes is a common strategy used by both pathogens (Durmuş Tekir et al., 2012). In addition to these comparative interactomics studies for bacterial and viral PHI networks, a comparative analysis of virus interactions with human signal transduction pathways revealed that different viruses tend to target the same cellular pathways, not necessarily via interacting with the same cellular proteins (Singh et al., 2010).

## Web-Based Databases for PHI Systems

In parallel with the first large-scale experimentally verified PHI data, the initial efforts on the development of PHI-specific databases were performed toward the end of the first decade of this century (**Table 2**). Currently, a number of Web-based resources aim to integrate pathogen–host molecular interactions and related data available in the literature. Some of them store data on only one specific pathogen species as in the case of HCVpro (Kwofie et al., 2011), HIV-1 Human Interaction Database at NCBI (Ako-Adjei et al., 2015), HoPaCI-DB (Bleves et al., 2014) for *Pseudomonas aeruginosa* and *Coxiella burnetii*, and Proteopathogen (Vialás et al., 2009) for *C. albicans*. The resources based on a wider range of specific pathogens are VirHostNet (Guirimand et al., 2015), VirusMentha (Calderone et al., 2015) and ViRBase (Li et al., 2015) for viruses, PATRIC (Wattam et al., 2014) for bacteria and PHI-base (Urban et al., 2015) for bacterial, fungal, and oomycete pathogens. Finally, PHIDIAS (Xiang et al., 2007), HPIDB (Kumar and Nanduri, 2010), and PHISTO (Durmuş Tekir et al., 2013) are PHI databases for all pathogen types with known interaction data.

**Table 2:** Contents of Web-based PHI databases.

Database	Number of PHIs	Pathogen	Host	Reference
HCVPro	524	Only HCV	Only human	Kwofie et al. (2011)
HIV-1 Human at NCBI	12,786	Only HIV-1	Only human	Ako-Adjei et al. (2015)
HoPaCI-DB	4203	*Pseudomonas aeruginosa* and *Coxiella burnetii*	Mammalia, *Caenorhabditis elegans*, *Drosophila Melanogaster*, *Danio rerio*	Bleves et al. (2014)
HPIDB	40,611	Bacteria, fungi, viruses	Animal, human, plant	Kumar and Nanduri (2010)
PATRIC	8547	Only bacteria	Actinopterygii, Arachnida, Chromadorea, Insecta, Mammalia	Wattam et al. (2014)
PHI-base	4102	Bacteria, fungi, oomycete	Animal, human, insect, fish, fungi, plant	Urban et al. (2015)
PHIDIAS	NA	Bacteria, viruses, parasites	All hosts	Xiang et al. (2007)
PHISTO	39,166	Bacteria, fungi, Protozoa, viruses	Only human	Durmuş Tekir et al. (2013)
Proteopathogen	NA	*Candida albicans*	Mammalia	Vialás et al. (2009)
ViRBase	NA	Only viruses	All hosts	Li et al. (2015)
VirHostNet	16,000	Only viruses	Animal, human, plant	Guirimand et al. (2015)
VirusMentha	8084	Only viruses	All hosts	Calderone et al. (2015)

HCVPro (HCV interaction database) is dedicated to only HCV, cataloging the characterized protein interactions for intraviral and virus–human systems. Additionally, it includes information on the structure and functions of HCV proteins (Kwofie et al., 2011). The HIV-1 Human Protein Interaction Database at NCBI includes the interactions between HIV-1 and human proteins. In its content, the majority of the protein interaction data are indirect (e.g., upregulation, modification) whereas the rest are direct (e.g., binding; Ako-Adjei et al., 2015). HoPaCl-DB (Host–Pseudomonas and Coxiella interaction database) provides information on interactions between molecules, bioprocesses, and cellular structures for the bacterial pathogens *Pseudomonas aeruginosa* and *C. burnetti* and their host organisms. The graphical representation of these interaction systems is also available in HoPaCl-DB (Bleves et al., 2014). The other pathogen-specific data resource, Proteopathogen is a protein database for studying *C. albicans*–host interactions. Although the focus of the database is on *C. albicans* and its interactions with macrophages, the database also includes data for different fungal pathogens and other mammalian cells. Proteopathogen provides additional information about the interacting proteins such as Gene Ontology (GO) and pathway annotations, and protein structures (Vialás et al., 2009).

PATRIC (The PathoSytems Resource Integration Center) is a dedicated resource for bacterial systems including comprehensive data on genomics, transcriptomics, PPIs, 3D protein structures, and sequence typing. However, its focus is on the genomic data, currently covering more than 10,000 bacterial genome sequences. PATRIC provides a private workspace for each user where they can store their own data. In their workspaces, users can perform comparative genomics and transcriptomics via the corresponding analysis tools. PATRIC provides bacteria–host PPI data through its tool Pathogen Integration Gateway (PIG; Wattam et al., 2014). PHI-base (Pathogen–Host Interactions Database) is a Web-accessible PHI database specific for bacterial, fungal, and oomycete pathogens, which are medically and agronomically important. PHI-base serves options to facilitate the discovery of genes that may be potential targets for chemical intervention, containing information on the pathogenicity/virulence genes functioning in the PHI systems. As a genomic data focused resource, PHI-base has the functionalities allowing functional annotations of the genes and comparative genomics analysis (Urban et al., 2015). On the other hand, there are databases developed specifically for viral PHI systems such as VirHostNet (Guirimand et al., 2015), VirusMentha (Calderone et al., 2015) and ViRBase (Li et al., 2015). VirHostNet (Virus–Host Network) is one of the earliest PHI resources specialized in the management and analysis of integrated virus–virus, virus–host, and host–host protein interaction networks coupled to their functional annotations. The host organism in the VirHostNet is only human. Its Web interface provides both table-based and graph-based visualizations of the PHI networks (Guirimand et al., 2015). The recently developed tool, VirusMentha is another virus-virus and virus–host protein interaction resource. VirusMentha is an extension of a previous tool VirusMINT (Chatr-Aryamontri et al., 2009). VirusMentha is the most comprehensive viral PHI data source without limitation with respect to virus species or host organisms. The tool offers a graphical representation option for viral PHI networks (Calderone et al., 2015). On the other hand, ViRBase is a resource for virus–host ncRNA-associated interactions. It provides browsing and visualization of viral and cellular ncRNA-associated virus–virus, host–virus, and host–host interactions (Li et al., 2015).

Finally, the Web-based PHI databases comprising all pathogen types with known interactions are PHIDIAS (Xiang et al., 2007), HPIDB (Kumar and Nanduri, 2010), and PHISTO (Durmuş Tekir et al., 2013). PHIDIAS (Pathogen–Host Interaction Data Integration and Analysis System) stores data on genome sequences, conserved domains, and gene expression data related to PHIs. In addition to data storage, PHIDIAS offers the analysis of these data (Xiang et al., 2007). HPIDB (Host–Pathogen Interaction Database) is not limited to any pathogen or host regarding pathogen–host PPI data. HPIDB offers the BLASTP search option that allows searching for homologous PHI data for pathogens without experimental PHI data (Kumar and Nanduri, 2010). Currently, PHISTO (Pathogen-Host Interaction Search Tool) is the most comprehensive PHI database on the Web including data for all pathogenic microorganisms for which experimental protein interactions with human are available. Bioinformatic analysis tools in PHISTO allow users to visualize and analyze PHI networks to get insights on infection mechanisms (Durmuş Tekir et al., 2013). Using the tools in the current version of PHISTO, users can access the functional and topological properties of pathogen-targeted human proteins within the human intranetwork. Furthermore, a comparative analysis tool is provided to perform these analyses comparatively for different pathogens to observe the similarities and differences in their infection strategies.

Pathogen–host protein interaction data in the above PHI databases are integrated mainly from other PPI databases using automatic integration tools such as PSICQUIC (Aranda et al., 2011) and by manual curation from the literature. For the PHI tools, commonly used PPI databases including PHI data are APID (Prieto and De Las Rivas, 2006), BIND (Alfarano et al., 2005), BioGrid (Chatr-aryamontri et al., 2013), DIP (Salwinski et al., 2004), HPRD (Keshava Prasad et al., 2009), IntAct (Orchard et al., 2013), iRefIndex (Razick et al., 2008), MINT (Licata et al., 2012), NetworKIN (Horn et al., 2014), Reactome (Croft et al., 2014), and STRING (Franceschini et al., 2013).

There are other informative databases for pathogens, providing useful information for studying infection mechanisms. For instance, ARDB (Antibiotic Resistance Genes Database) unifies most of the publicly available information on antibiotic resistance. The information can be used as a compendium of antibiotic resistance genes of newly sequenced genomes (Liu and Pop, 2009). IVDB (Influenza Virus Database) is an integrated information resource and analysis platform for influenza virus research focusing on the genetic, genomic, and phylogenetic studies. IVDB provides complete genome sequences of the virus to facilitate the analysis of global viral transmission and evolution (Chang et al., 2007). MPIDB (Microbial Protein Interaction Database) aims to collect all known physical interactions among the bacterial proteins (Goll et al., 2008). MvirDB is a microbial database of protein toxins, virulence factors, and antibiotic resistance genes for bio-defense applications (Zhou et al., 2007). VFDB (Virulence Factor Database) is a comprehensive repository for bacterial virulence factors (Chen et al., 2011). VIDA is a virus database system for open reading frames (ORFs) of animal viruses (Albà et al., 2001). Finally, ViPR (Virus Pathogen Database and Analysis Resource) is an open bioinformatic resource for virology research. ViPR captures various types of information, including sequence data, gene, and protein annotations, 3D protein structures, clinical and surveillance metadata, and novel data derived from comparative genomics analyses (Pickett et al., 2012).

## Text Mining of PHI Data from the Literature

Scientific publications are the main media through which researchers report their new findings. The huge amount and the continuing rapid growth of the number of published articles in biomedicine has made it particularly difficult for researchers to access and utilize the knowledge contained in them. Currently, there are over 24 million publications indexed in PubMed^11^, which is the main system that provides access to the biomedical literature.

To address the challenge of information overload in the biomedical literature, a number of manually curated databases have been developed to store biologically important information such as protein interactions, gene–disease associations, or PHIs. However, given the current amount and the continuing rapid growth of the biomedical literature, it usually takes a lot of time and effort before new discoveries are included in these databases. Human database curation cannot keep up with literature production (Baumgartner et al., 2007). As a consequence, most of the knowledge remains hidden in the unstructured text of theh publised articles. Therefore, developing text mining techniques to uncover this knowledge has become an important research area. Several text mining approaches have been proposed for identifying articles relevant to a particular topic, detecting biomedical entities such as genes, proteins, and diseases in text, as well as extracting the relations among them. A number of shared tasks such as the BioCreative Challenges (Krallinger et al., 2008; Arighi et al., 2011) and the BioNLP Shared Tasks (Kim et al., 2009, 2011; Nédellec et al., 2013) have been conducted, which have further boosted research in this area. However, text mining for the pathogen-host interactions domain has not been well studied yet, although it has its own peculiarities and challenges. Only a handful of studies, which are discussed in the subsections below, have been conducted so far in this domain. One thread of research focuses on identifying the articles that contain PHI-relevant information (Yin et al., 2010; Korkin et al., 2011; Thieu et al., 2012) and another thread of research addresses performing more detailed semantic analysis of the text and extracting more fine-grained information such as the specific proteins that interact and the associated pathogen and host organisms (Korkin et al., 2011; Thieu et al., 2012).

### PHI-Relevant Abstract Detection

Identifying and ranking articles that contain PHI-relevant information can be used for selecting and prioritizing articles for manual curation. It can also be an initial step for filtering the relevant articles before performing more fine-grained semantic analysis for identifying the biomedical entities and the relations among them. The task for detecting articles describing PPI information has been addressed in the BioCreative II, II.5, and III challenges (Krallinger et al., 2008; Leitner et al., 2010; Arighi et al., 2011). However, the focus has not been on PHI relevant articles. The first study that focused on detecting PHI-relevant abstracts, i.e., abstracts that describe pathogen host PPI, was conducted by (Yin et al., 2010). Similarly to most systems that participated in the BioCreative Challenges Article Classification Task, the problem was formulated as a supervised machine learning based classification task. Support Vector Machines (SVM) was used as the classification algorithm (Cortes and Vapnik, 1995). Feature selection methods including Information Gain, Mutual Information, and Chi-square were evaluated using a data set of 1360 manually labeled abstracts. The results showed that Information Gain and Chi-square perform better than Mutual Information as the number of features used decreases. Although the focus of the study was on PHI-relevant abstract classification, no any PHI specific features were used. Only the word unigrams and bigrams were used as features.

Pathogen–host interaction-relevant abstract classification was also tackled by (Thieu et al., 2012). Similarly to (Yin et al., 2010), the task was addressed as a supervised machine learning classification problem and SVM was used as the classification algorithm. However, unlike (Yin et al., 2010), the authors defined and used PHI specific features including the identified host and pathogen protein and gene names in the text, the host and pathogen organism names, the interaction signaling keywords, the experimental method keywords, and PHI-specific keywords such as virulence and effector. In order to account for the abstracts that report the absence of an interaction between a host and pathogen protein, features that make use of the negation signaling keywords were also designed. The protein and gene names, as well as the corresponding organisms were tagged by using the NLProt software (Mika and Rost, 2004). A set of dictionaries for interaction keywords, experimental keywords, negation keywords, PHI-keywords, host names, pathogen names, and uncertainty keywords was manually compiled. A data set of 175 PHI-relevant (positive set) and 175 PHI non-relevant (negative) abstracts was manually annotated and used for evaluation. The results showed that using PHI specific features is a promising approach for identifying PHI-relevant articles. However, it is not possible to compare the results with the results of (Yin et al., 2010), since a different data set was used for evaluation.

In order to be able to assess the performances of the proposed methods a larger and publicly available benchmark data set should be created. Such a data set should in fact contain three types of abstracts: (1) Abstracts that do not contain any PPI information (negative class 1); (2) Abstracts that contain PPI information which are not pathogen–host PPIs (negative class 2); and (3) Abstracts that contain pathogen–host PPI information (positive class). Distinguishing the positive class from negative class 2 is probably more difficult, since they both contain PPI information. The only difference is that the PPIs in negative class 2 are not PHIs. To distinguish these two classes from each other, PHI specific features should be utilized. On the other hand, distinguishing the positive class from negative class 1 is probably easier and generic PPI relevant features might be sufficient. It is not clear whether the data sets annotated and used in Yin et al. (2010) and Thieu et al. (2012) contain these three classes, or contain only two of them (i.e., the positive class and negative class 1). Therefore, it is difficult to assess and compare the reported results.

### PHI-Relevant Relation Extraction

One of the most important opportunities for text mining in biomedicine is the identification of the relations among the biomolecules, which can help elucidate their roles in important biological processes, as well as in diseases. In order to extract the relations among biomedical entities from text, first the sequences of characters that correspond to entities should be tagged in text. This task is called Named Entity Recognition (NER) and has been an active research topic in the biomedical text mining domain.

While the earliest systems for biomedical NER were usually based on rule-based approaches (Fukuda et al., 1998), as annotated corpora became available, machine-learning based methods gained popularity (McDonald and Pereira, 2005; Tsai et al., 2006; Hsu et al., 2008). State-of-the-art gene and protein NER systems achieve a practically applicable level of performance (e.g., 87% *F*-score performance was obtained at the second BioCreative shared task on gene mention tagging (Smith et al., 2008)). Genia Tagger (Tsuruoka et al., 2005), ABNER (Settles, 2005), and BANNER (Leaman and Gonzalez, 2008) are some of the publicly available biomedical NER tools. LINNAEUS (Gerner et al., 2010) and OrganismTagger (Naderi et al., 2011) are tools developed for recognizing species names in biomedical text. Both achieve *F*-score performances of over 94%. Although the usability of these NER tools for the PHI domain has not been well addressed yet, in principle they can also be used for PHI text mining to identify the entity names such as gene, protein, and species names in text.

One of the first studies on using text mining for pathogen–host relationship extraction was conducted by (Anthony et al., 2010). As a case-study, the authors targeted the extraction of genotype, pathogen, and syndrome relations. A corpus consisting of 43 abstracts from PubMed was manually annotated. The available technologies for the automatic recognition of host–pathogen named entities and the relations among them were discussed. However, they have not been evaluated over the annotated corpus, which makes it difficult to draw conclusions about their usability for the PHI text mining domain.

Thieu et al. (2012) addressed the problem of extracting pathogen–host PPIs from text. The authors proposed a linguistically motivated approach that makes use of the link grammar representations of the sentences (Sleator and Temperley, 1995). Thieu et al. (2012) generated additional rules to map the protein names to the corresponding pathogen and host organism names. For instance, if an organism name occurs before a protein name (e.g., *Arabidopsis* RIN4 protein) the protein is mapped to the preceding organism. In addition, Thieu et al. (2012) incorporated an anaphora resolution module that resolves the pronouns such as “it,” “they,” etc. in the sentences with their corresponding protein/gene or organism names, which makes possible extracting relations that span multiple sentences. This module is based on the RelEx anaphora resolution method that uses the Hobbs’ pronoun resolution algorithm (Hobbs, 1978). The proposed approach was evaluated by using the 350 annotated abstracts described in the section “PHI-Relevant Abstract Detection.” The results of (Thieu et al., 2012) showed that the proposed approach significantly outperformed a naïve approach based on using one of the state-of-the-art generic PPI extraction tools Protein Interaction information Extraction (PIE) system (Kim et al., 2008). This motivates the development of methods that specifically address pathogen–host PPI extraction. The 24% *F*-score obtained by the proposed system suggests that there is room for improvement and further research in this domain is necessary. An error analysis suggested that an important source of error was the incorrect identification of protein names and incorrect assignment of species to the corresponding proteins. While the first one is a NER problem, which is an active research topic in biomedical text mining, the second one has not been tackled much by the researches. The results of the current studies suggest that it is a crucial research direction for PHI text mining studies.

Pathogen–host interaction-specific PPI extraction is a similar problem to the general problem of mining PPI relevant information from text (Ono et al., 2001; Blaschke and Valencia, 2002; Temkin and Gilder, 2003; Daraselia et al., 2004; Jelier et al., 2005; Erkan et al., 2007; Fundel et al., 2007; Airola et al., 2008; Tikk et al., 2010). However, it has its own peculiarities that require the development of methods specialized for PHI text mining. In order for a PPI to qualify as a PHI, the interaction should be intra-species. In other words, one of the proteins should be a host protein and the other one should be a pathogen protein. Therefore, besides tackling the problem of extracting the pair of proteins that interact, the problems of identifying the species associated with them, as well as the classification of the species as host or pathogen should also be addressed. These additional requirements render the PHI text mining task more difficult than the already challenging PPI text mining task. Most PPI extraction systems operate on a sentence-level to extract the interactions. The underlying assumption is that the majority of the relations are contained within a single sentence. Analysis of the Genia event corpus (Kim et al., 2009) supports this assumption, since only 5% of the relations in the corpus span multiple sentences (Björne et al., 2009). However, this assumption does not in general hold for the PHI extraction task, since in many cases the species of the associated entities do not occur in the same sentence where the interaction is described (Thieu et al., 2012). Therefore, in order to extract PHIs from text, wider scope than a sentence should be considered and methods to merge information contained in multiple sentences should be developed. Nevertheless, the current findings from the generic PPI text mining domain can be utilized. For instance, recent studies have demonstrated the utility of integrating machine learning methods with similarity functions (or kernels) defined using the syntactic and semantic analysis of text (Tikk et al., 2010). Some of these approaches can be adapted to the PHI text mining domain by performing anaphora resolution as a prior step and extending the methods to operate on scopes wider than a sentence. In addition, novel methods should be developed to address the problem of assigning the species to their corresponding entities (e.g., proteins and genes). Sentence-level processing will probably not be sufficient to develop solutions to this problem, since species names do not necessarily occur in the same sentences or even in the same paragraphs as the entity names. Another challenge is that a species can be a host in one context, while it is a pathogen in another context. Therefore, methods for determining which species are pathogens and which are host in the given context should be designed.

The PHI information extracted using text mining can be utilized in at least two ways. First, such information can be used to populate PHI databases, either directly or indirectly by facilitating manual curation. This will make the data buried in the literature easily accessible to the researchers in this domain. Second, further analysis of the uncovered information can be integrated into a systems biology approach to generate new scientific hypothesis such as predicting currently unknown interactions among pathogen and host proteins.

## Conclusion and Future Directions

Conventional therapeutics aim to kill pathogenic microorganisms directly usually by targeting the pathogen only. However, the drug resistance of pathogens demands alternative solutions for infectious threats, i.e., targeting host proteins required by pathogens for replication and persistence within the host organism or targeting PHIs (Murali et al., 2011; Zoraghi and Reiner, 2013). If these host proteins are indispensable for pathogens during infections, but not essential for host cells, they may serve as antimicrobial therapeutic targets to fight drug resistance. In parallel with the increase in the amount of PHI data, several genome-wide RNAi screening studies to identify cellular host factors were performed within the last decade (Ng et al., 2007; Brass et al., 2008; Hao et al., 2008; König et al., 2008, 2010; Krishnan et al., 2008; Zhou et al., 2008; Bushman et al., 2009; Li et al., 2009; Sessions et al., 2009; Tai et al., 2009; Karlas et al., 2010; Kumar et al., 2010; Murali et al., 2011; Moser et al., 2013; Lee et al., 2014). The detailed knowledge about mechanisms of the relationships between these host factors and their targeting pathogens is required urgently to develop new and more effective antimicrobial therapeutics, necessitating a computational systems biology approach to PHIs.

The computational modeling of networks of interacting genes, transcripts, proteins, and metabolites is of great importance in biomedical research to understand molecular mechanisms of PHIs. The high-throughput experimental detection of levels of biomolecules (gene transcripts, proteins, and metabolites) via omics approaches as well as the detection of PHIs via high-throughput experiments has generated comprehensive datasets. The presented review has provided a snapshot of recent developments in this area and a survey about databases that store such infection-specific data. Using text mining is necessary to extract the PHI-relevant data that are only available in the text of the huge amount of scientific literature. Although biomedical text mining is an active research area, there are only a limited number of studies focusing on extracting PHI information. The lack of a publicly available data set (‘gold standard’) makes it difficult to evaluate and compare the current approaches. Besides reviewing the current studies, we have also provided future directions for research including analyzing the usability of the already available biomedical text mining methods for the PHI text mining task, developing novel approaches addressing the peculiarities and challenges of the PHI domain, and creating publicly available benchmark data sets in order to provide a better assessment of the different methods. We have also covered studies on the bioinformatic analysis of three types (protein-based, regulatory, and metabolic) of PHI networks. The integrative analysis of the high-throughput omics experiments using modeling approaches will not only elucidate the mechanisms of infection, but will help in the discovery of potential therapeutic targets and drugs through selective identification of essential genes, proteins, and metabolites for the pathogen. Despite the recent efforts reviewed above, the use of systems biology approaches to investigate PHI systems is still in its infancy, mostly because of data scarcity. Ongoing studies in the field will lead to more complete PHI networks in the coming decade, improving the PHI-based solutions to infectious diseases.

## Acknowledgments

A was supported by Marie Curie FP7-Reintegration-Grants within the 7th European Community Framework Programme. RG was supported by the Deutsche Forschungsgemeinschaft (DFG) in the Collaborative Research Centre/Transregio 124 FungiNet (subprojects B3 and INF).

## Conflict of Interest Statement

The authors declare that the research was conducted in the absence of any commercial or financial relationships that could be construed as a potential conflict of interest.

## Footnotes

1 http://fged.org

2 http://www.sysmo-db.org

3 http://www.omnifung.hki-jena.de

4 http://bioconductor.org

5 http://www.cs.waikato.ac.nz/ml/weka

6 http://www.rapidminer.de

7 https://gold.jgi-psf.org

8 http://www.ncbi.nlm.nih.gov/geo

9 https://www.ebi.ac.uk/arrayexpress

10 http://www.the-dream-project.org

11 http://www.ncbi.nlm.nih.gov/pubmed
